# A dataset on forest stand structures, deadwood, and tree-related microhabitats along an urban-periurban gradient in Central Italy

**DOI:** 10.1016/j.dib.2025.111561

**Published:** 2025-04-15

**Authors:** Costanza Borghi, Soraya Versace, Elena Di Pirro, Davide Travaglini, Gherardo Chirici, Bruno Lasserre, Marco Marchetti, Giovanni D’Amico, Elia Vangi, Saverio Francini, Marco Montella, Giovanni Santopuoli, Marco Ottaviano, Francesco Parisi

**Affiliations:** a geoLAB - Laboratorio di Geomatica Forestale, Dipartimento di Scienze e Tecnologie Agrarie, Alimentari, Ambientali e Forestali, Università degli Studi di Firenze, Via San Bonaventura 13, 50145 Firenze, Italy; b Fondazione per il Futuro delle Città, Firenze, Italy; c Department of Bioscience and Territory, University of Molise, Contrada Fonte Lappone, 86090 Pesche, Italy; d NBFC, National Biodiversity Future Center, Palermo 90133, Italy; e Department of Architecture and Design, Sapienza University of Rome, Via Flaminia, 359, 00196 Rome, Italy; f Dipartimento di Scienze e Tecnologie Agro-alimentari (DISTAL), Università di Bologna, 40126 Bologna, Italy; g Dipartimento di Agricoltura Ambiente ed Alimenti, Università degli Studi del Molise, via F. de Sanctis, 86100 Campobasso, Italy; h Forest Modelling Lab., Institute for Agriculture and Forestry Systems in the Mediterranean, National Research Council of Italy (CNR-ISAFOM), Via Madonna Alta, 128, Perugia, Italy

**Keywords:** Urban forest, Urban ecology, Urban biodiversity, Dead tree, Urban park, Monumental garden, Monumental tree, City

## Abstract

This database provides accessible and georeferenced information on forest structure, tree-related microhabitats, and deadwood of 12 urban forests located in 12 different urban parks across three Italian cities, Florence, Rome, and Campobasso. Four urban parks – varying in size, forest type, and history – were selected following an urban-periurban gradient in each city. Inner city parks are typically ancient, with native and non-native trees planted for aesthetic and cultural purposes, and scarce semi-natural vegetation remains. Periurban parks usually host native and semi-natural vegetation and may include agricultural areas. 15 plots were placed to survey a selected urban forest located in each of the 12 urban parks, using a systematic aligned sampling scheme and then visited in the field, for a total of 180 plots. The collected data contributed to the construction of three different datasets. Two tree-level datasets present information on tree-related microhabitats and dendrometric variables including tree species, diameter at breast height, tree height, height-to-base of the live crown, tree volume, and tree basal area. The deadwood dataset presents information on five categories of deadwood, particularly snags, standing dead trees, coarse woody debris, stumps, and dead downed trees, where height, diameter, and decay status were sampled. Other research can employ these data to integrate and compare databases from different cities and forest types. Additionally, data can be linked to future analyses of urban forest fauna (e.g., beetle and bird communities) and updated to assess variability over time as well as employed in landscape analysis to guide improved management actions.

Specifications Table

**Table:** 

Subject	Earth and Environmental Sciences
Specific subject area	Dendrometric measurement; living trees, deadwood, and tree-related microhabitats survey
Type of data	Table, Georeferenced data Raw, Analyzed
Data collection	Datasets are based on *in-situ* measurements conducted within 180 circular sample plots, each with a radius of 13 meters. Plots were distributed across twelve urban and periurban forests and parks (four for each location, spanning a gradient from urban to periurban environments) in three Italian urban areas: Florence, Rome, and Campobasso (Table 1). The data collection focused on living trees, deadwood, and tree-related microhabitats, following standardized sampling protocols. The instruments used for data acquisition included a tree calliper, GNSS, hypsometric equipments and an ultrasonic meter (i.e, Vertex).
Data source location	Three Italian Municipalities (NUTS3 level): Florence, Rome, Campobasso
Data accessibility	Repository name: Figshare Data identification number: 10.6084/m9.figshare.28450643 Direct URL to data: https://figshare.com/articles/dataset/Urban_and_Periurban_Forests_of_Italy_Structure_Tree-related_Microhabitats_and_Deadwood/28450643

## 1 Value of the Data

• Dataset can be used to assess broadleaves forest stand structures and microhabitat typologies along an urban-periurban gradient across different urban contexts.
• Collected data contributes to increasing knowledge about deadwood and microhabitats as ecological indicators, usually scarcely addressed in urban environments.
• Data can be employed to build a more comprehensive dataset including multiple cities from different climatic zones and forest types.
• Locations and data can be related to landscape and context analysis to guide management actions on urban biodiversity.
• Collected data can be integrated with an investigation on multi-taxon communities to evaluate their state-of-the-art in urban contexts.
• Data can be combined with other sources of data including remotely sensed data to build new models and to improve urban green areas and biodiversity monitoring.

## 2 Background

Thanks to their proven capacity to provide ecosystem services, urban forests and trees are increasingly assessed, implemented, and maintained across different urban contexts [4]. Despite their role in contributing to climate regulation, air and water quality, and recreation and cultural activities being largely evaluated and discussed in the literature, their capacity to support biodiversity is still little known. Tree-related microhabitats and deadwood are two critical ecological indicators frequently employed in forestry studies to estimate species abundance and richness [18]. In natural forests, the limitation of deadwood resources can alter or threaten biodiversity, as its various forms and stages of decay are important for organisms’ existence (e.g., saproxylic beetles, [2]) and provide diverse ecosystems' functions [6]. Furthermore, different studies [16] showed evidence of the relationship between forest stand structure, management, and biodiversity support service. However, research expanding this methodological approach and data collection to the urban environment is still scarce in the literature. This dataset wants to contribute to filling this knowledge gap.

## 3 Data Description

**Fig. 1:**
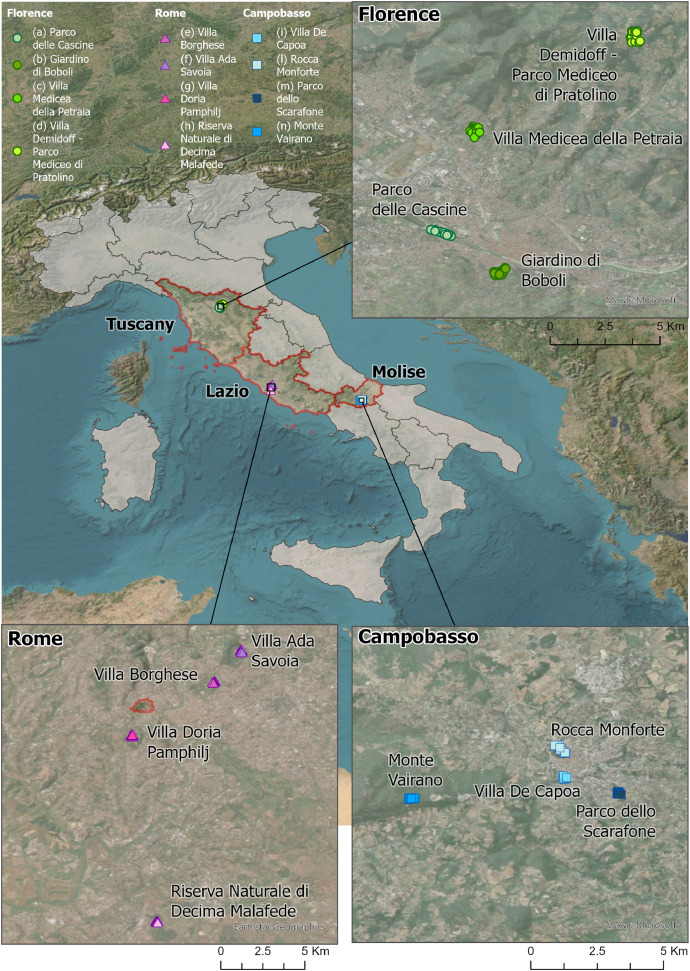
180 sampled plots placed to survey urban forests in each of the 12 urban parks across the three urban areas and related administrative regions, Florence (Tuscany region), Rome (Lazio region) and Campobasso (Molise region).

This article describes the database of the linked repository of “Urban and Periurban Forests of Italy: Structure, Tree-related Microhabitats, and Deadwood” [1], showing data collected between 2022 and 2025 across 12 different urban forests as portions of respective 12 urban parks in three Italian urban areas, Florence, Rome, and Campobasso (Fig. 1). Coordinates and locations are provided in Table 1 and refer to the centroid of the urban forest (WGS-84 reference system), while specific locations of the 15 sampling plots within the surveyed forests will be made available on request.

Specifically, eight different files are provided in the repository, (i) the metadata (in xlsx format), (ii-iii) two tree-level datasets (the former regarding the plot structure, the latter tree-related microhabitat, both in xlsx format), (iv) and a deadwood dataset (related to five categories of deadwood, reported and divided into 5 sheets, in xlsx format), and (v) the georeferenced centroids of the sampled urban forests (WGS84 reference system, gpkg format), whose attributes are available in Table 1. Moreover, for each dataset (i.e., forest structure, tree-related microhabitats, and deadwood) an R code for basic elaborations and graphs’ creation was provided (R v. 4.4.3). The two tree-level datasets are organized into 4508 rows and 12 columns and 4148 rows and 72 columns, respectively. In the tree-level datasets, the rows represent the instances, and the columns represent the information collected for each tree. Table 2 shows an example of the first dataset providing the information on location, tree species, and forest structure (i.e., tree species, diameter at breast height, tree height, height-to-base of live crown, tree volume, and tree basal area), and Table 3 shows an example of the second dataset providing information on the tree-related microhabitats collected and classified according to the catalogue of Tree-related Microhabitats by Kraus et al. [9]. The deadwood dataset provides information related to five categories of deadwood and it is organized according to five sheets, particularly Coarse Woody Debris (CWD, sheet 3), Standing Dead Trees (SDT, sheet 4), Stumps (sheet 5), Snags (sheet 6) and Dead Downed Trees (DDT, sheet 7). Table 4 shows all the parameters collected for each category of deadwood in each plot across the case studies areas.

**Table 1:** Details of the 12 urban and periurban forests in the dataset.

Urban Parks	Parco delle Cascine	Giardino di Boboli	Villa Medicea della Petraia	Villa Demidoff - Parco Mediceo di Pratolino	Villa Borghese	Villa Ada Savoia	Villa Doria Pamphilji	Riserva Naturale di Decima Malafede	Villa De Capoa	Rocca Monforte	Parco Scarafone	Monte Vairano
Acronym	CAS	BOB	LPE	DEM	BOR	ADA	PAM	MAL	DCA	MON	SCA	MVA
Municipality (study area)	Florence	Florence	Florence	Florence	Rome	Rome	Rome	Rome	Campobasso	Campobasso	Campobasso	Campobasso
Region (NUT2)	Tuscany	Tuscany	Tuscany	Tuscany	Lazio	Lazio	Lazio	Lazio	Molise	Molise	Molise	Molise
Coordinates N (decimals)	43.779274 903775836	43.762634 52408981	43.820133 1780011	43.859344 08642874	41.916350 83900851	41.932774 71171798	41.887797 68474425	41.788098 0657799	41.5546996 2280234	41.56465 797601508	41.54950 768131647	41.54772 4232210335
Coordinates E (decimals)	11.224590 824561696	11.248936 470714806	11.238763 893730656	11.304736 781520333	12.490593 519298729	12.505485 400016385	12.447023 146048544	12.460323 595163992	14.656808 932450526	14.655281 388658103	14.675150 515998343	14.605073 282990729
Altitude (m a.s.l.)	42	76	134	435	54	24	77	52	703	726	607	901
Number of sampling plots	15	15	15	15	15	15	15	15	15	15	15	15
Natura 2000 site code	-	-	-	-	IT6030052	-	IT6030052	-	-	IT7222125	-	IT7222295
Sampled area (ha)	0.8	0.8	0.8	0.8	0.8	0.8	0.8	0.8	0.8	0.8	0.8	0.8
Urban forest area (ha)	16.009	22.849	19.092	9.878	2.029	2.293	2.58	1.1823	1.52	4.468	1.353	1.09
Percentage sampled (%)	5.0	3.5	4.2	8.1	39.4	36.4	31.0	67.7	52.6	17.9	59.1	73.4

**Table 2:** Tree-level dataset description, location, species, and forest structure of living trees.

Site	acronym	plot_ID	N2k_sitecode	ID_tree	sp_tree	dbh_tree (cm)	h_tree (m)	h_INS_tree (m)	V_tree (m^3^)	BA_tree (m^2^)
Name of the site	Acronym of the site	Identification number for each plot, per site: “*acronym* _ *number of the plot”*	Natura 2000 site code (if applicable)	Identification number per each tree in each plot	Tree species (latin name)	Diameter at breast height	Tree height	Height-to-base of live crown	Tree volume	Tree basal area

**Table 3:** Tree-related microhabitats classified for each tree according to the definition of the Catalogue of Tree Microhabitats by Kraus et al. [9].

(Sheet 2) Tree-related microhabitats [*]		
Form	Head	Definition
CV1- Woodpecker cavities	CV11	Cavity entrance about ø = 4 cm
	CV12	Cavity entrance about ø = 5–6 cm
	CV13	Cavity entrance width is about ø > 10 cm
	CV14	Cavity entrance width is about ø ≥ 10 cm
	CV15	At least three in the trunk connected woodpecker breeding cavities
CV2- Trunk and mould cavities	CV21	Mould containing trunk cavity with ground contact about ø ≥ 10 cm
	CV22	Mould containing trunk cavity with ground contact about ø ≥ 30 cm
	CV23	Mould containing trunk cavity without ground contact about ø ≥ 10 cm
	CV24	Mould containing trunk cavity without ground contact about ø ≥ 30 cm
	CV25	Semi-open trunk cavity with or without mould about ø ≥ 30 cm
	CV26	Large trunk cavity with open top and with or without ground contact about ø ≥ 30 cm
CV3- Branch holes	CV31	Rot-holes originating from branch breakage at trunk about ø ≥ 5 cm
	CV32	Rot-holes originating from branch breakage at trunk about ø ≥ 10 cm
	CV33	Hollow more or less horizontal branch about ø ≥ 10 cm
CV4- Dendrotelms and water-filled holes	CV41	Cup-shaped concavities to trunk base with the entrance diameter about ø ≥ 3 cm
	CV42	Cup-shaped concavities to trunk base with the entrance diameter about ø ≥ 15 cm
	CV43	Cup-shaped concavities to crown with the entrance diameter about ø ≥ 5 cm
	CV44	Cup-shaped concavities to crown with the entrance diameter about ø ≥ 15 cm
CV5- Insect galleries and bore holes	CV51	Gallery with single small bore holes
	CV52	Gallery with single large bore hole about ø ≥ 2 cm
IN1- Bark loss/exposed sapwood	IN11	Bark loss 25–600 cm^2^ and decay stage < 3
	IN12	Bark loss > 600 cm^2^ and decay stage < 3
	IN13	Bark loss 25–600 cm^2^ and decay stage = 3
	IN14	Bark loss > 600 cm^2^ and decay stage = 3
IN2- Exposed heartwood/trunk and crown breakage	IN21	Broken trunk with the diameter at the end about ø ≥ 20 cm
	IN22	Broken tree crown/fork with exposed wood ≥ 300 cm^2^
	IN23	Broken limb with the diameter at the end about ø ≥ 20 cm
	IN24	Splintered stem with the diameter at the end about ø ≥ 20 cm
IN3- Cracks and scars	IN31	Cleft through the bark with length ≥ 30 cm, width > 1 cm, depth > 10 cm
	IN32	Cleft through the bark with length ≥ 100 cm, width > 1 cm, depth > 10 cm
	IN33	Bark loss and crack caused by lightning
	IN34	Fire scars at the lower trunk ≥ 600 cm^2^
BA1- Bark pockets	BA11	Bark shelter with width > 1 cm, depth > 10 cm, height > 10 cm
	BA12	Bark pocket with width > 1 cm, depth > 10 cm, height > 10 cm
BA2- Bark structure	BA21	Coarse bark
DE1- Dead branches and limbs/crown deadwood	DE11	Decaying wood ø 10–20 cm, cm, ≥ 50 cm, sun exposed
	DE12	Decaying wood ø > 20 cm, ≥ 50 cm, sun exposed
	DE13	Decaying wood ø 10–20 cm, ≥ 50 cm, not sun exposed
	DE14	Decaying wood ø > 20 cm, ≥ 50 cm, not sun exposed
	DE15	Decaying wood with dead top ø ≥ 10 cm
GR1- Root buttress cavities	GR11	Natural cavity formed by the tree roots ø ≥ 5 cm
	GR12	Natural cavity formed by the tree roots ø ≥ 10 cm
	GR13	Trunk cleavage with length ≥ 30 cm
GR2- Witches broom	GR21	Dense agglomeration of twigs ø > 50 cm
	GR22	Dense agglomeration of shoots on the trunk or branches of a tree
GR3- Cankers and burrs	GR31	Cancerous growth ø > 20 cm
	GR32	Decayed canker ø > 20 cm
EP1- Fruiting bodies fungi	EP11	Annual polypores ø > 5 cm
	EP12	Perennial polypores ø > 10 cm
	EP13	Pulpy agaric ø > 5 cm
	EP14	Large ascomycetes ø > 5 cm
EP2- Myxomycetes	EP21	Myxomycetes ø > 5 cm
EP3- Epiphytic crypto- and phanerogams	EP31	Epiphytic bryophytes coverage > 25 %
	EP32	Epiphytic foliose and fruticose lichens coverage > 25 %
	EP33	Lianas coverage > 25 %
	EP34	Epiphytic ferns > 5 fronds
	EP35	Mistletoe
NE1- Nests	NE11	Large vertebrate nest ø > 80 cm
	NE12	Small vertebrate nest ø > 10 cm
	NE21	Invertebrate nest
OT1- Sap and resin run	OT11	Sap flow > 50 cm
	OT12	Resin flow and pockets > 50 cm
OT2- Microsoil	OT21	Crown microsoil
	OT22	Bark microsoil

**Table 4:** Deadwood dataset description, including five categories of deadwood (respectively sheets 3, 4, 5, 6, and 7 of the dataset). The deadwood decay stage was assessed through Hunter’s classification system (Hunter, 1990).

(Sheet 3) CWD - Coarse Woody Debris									
acronym	plot_ ID	ID_ CWD	Dmin_cm	Dmax_cm	length_m	species	decay_stage	V_m^3^	
Acronym of the site	N° of the plot, per each site	Identification number per CWD in each plot	Minimum diameter of the CWD	Maximum diameter of the CWD	Length of the CWD	Tree species (latin name) or NA (not detected)	Decay stage (1-5)	Volume of the CWD	
**(Sheet 4) SDT - Standing Dead Tree**									

**acronym**	**plot_ ID**	**ID_ SDT**	**dbh_ cm**	**h_m**	**species**	**decay_stage**	**V_m^3^**		

Acronym of the site	N° of the plot, per each site	Identification number per SDT in each plot	Diameter at breast height per each stump in each plot	Height per each SDT in each plot	Tree species per each SDT in each plot	Decay stage (1-5)	Volume per each SDT in each plot		
**(Sheet 5) Stumps**									

**acronym**	**plot_ ID**	**ID_ stump**	**Origin (N/A)**	**Dbase_cm**	**Dtop_cm**	**h_m**	**species**	**decay_stage**	**V_m^3^**

Acronym of the site	N° of the plot, per each site	Identification number per stump in each plot	Origin of the stump, Natural or Artificial	Base diameter of each stump	Top diameter of each stump	Height of each stump	Species of the stumps	Decay stage (1-5)	Volume of each stump in each plot
**(Sheet 6) Snags**									

**acronym**	**plot_ ID**	**ID_ snag**	**Dtop_cm**	**Dbase_cm**	**h_m**	**species**	**decay_stage**	**V_m^3^**	

Acronym of the site	N° of the plot, per each site	Identification number per snag in each plot	Top diameter of each snag in each plot	Top diameter of each snag in each plot	Height of each snag in each plot	Specie of each snag in each plot	Decay stage (1-5)	Volume of each snag in each plot	
**(Sheet 7) DDT - Dead Downed Trees**									

**acronym**	**plot_ ID**	**ID_ DDT**	**dbh_cm**	**length_m**	**species**	**decay_stage**	**V_m^3^**		

Acronym of the site	N° of the plot, per each site	Identification number per DDT in each plot	Diameter at breast height of each DDT in each plot	Length of each DDT in each plot	Species of each DDT in each plot	Decay stage (1-5)	Volume of each DDT in each plot		

## 4 Materials and Methods

### 4.1 Study areas and sampled sites

**Fig. 2:**
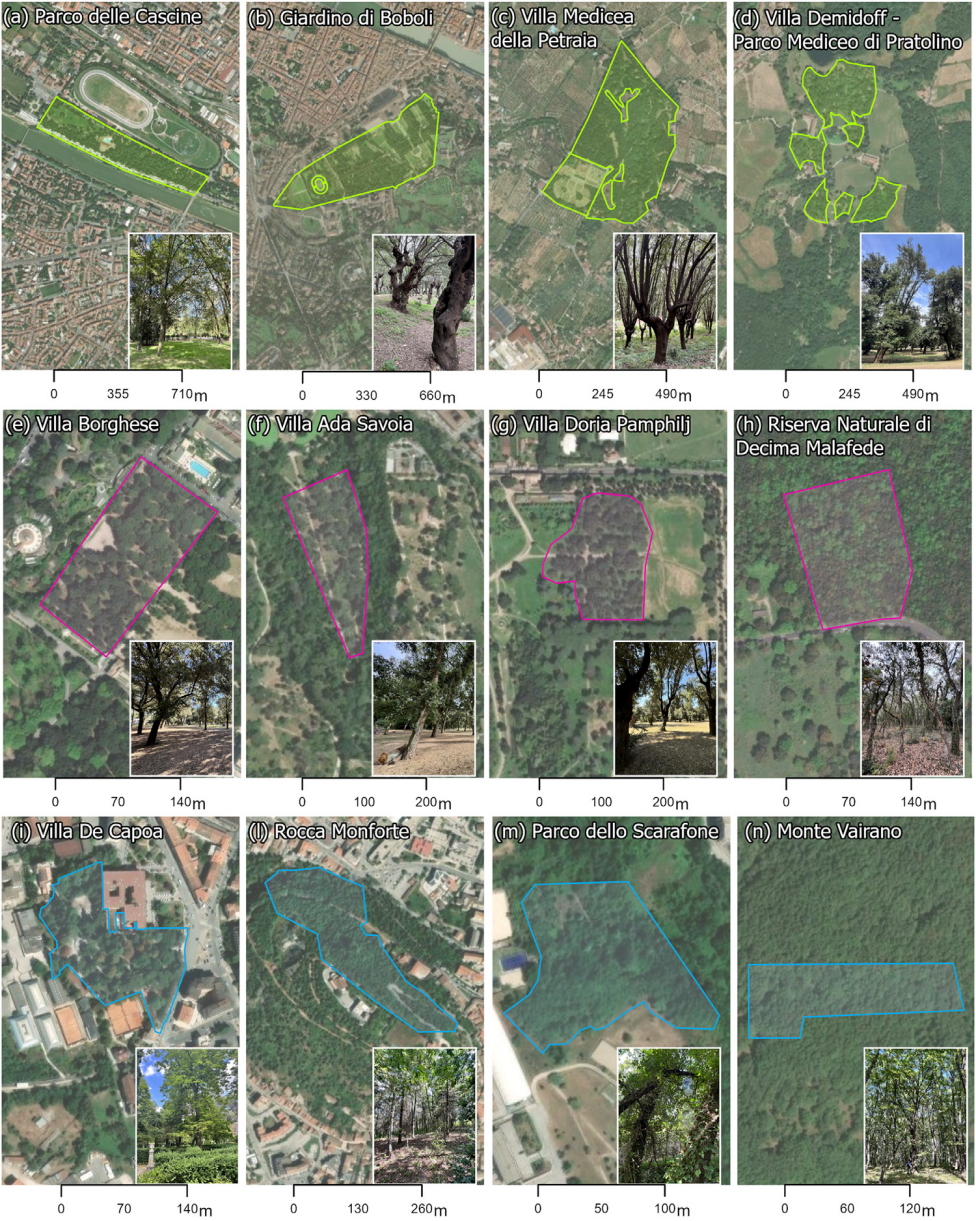
12 Urban and periurban forests surveyed within the 12 selected parks. a-d, Florence, e-h, Rome, and i-n, Campobasso.

Three cities have been selected for this data collection: Florence (102.32 km^2^, 363,000 inhabitants), Rome (1287.36 km^2^, 2,748,000 inhabitants), and Campobasso (56.11 km^2^, 48,000 inhabitants), located in central Italy. Following an urban-periurban gradient, four parks with different dimensions, morphology, and vegetational characteristics were selected in each city. The gradient was determined here as the Euclidean distance between the centroids of the urban parks and the urban centres identified by the Italian National Institute of Statistics (ISTAT) for 2021. According to Fig. 2, the history of the parks and the related vegetation are briefly introduced following the gradient from the city centre to the periurban area. For each park, one urban forest – partially covering the park area – was surveyed, and the main information retrieved on their current and past management are presented. However, management plans for urban forests are usually not disclosed; in particular, the history and provenance of plantations, the forest's natural evolution, and related maintenance activities are usually lacking. Therefore, the information refers to a collection of documents together with the evaluation carried out in the field by the operators. The urban forests analyzed were classified as “intensively managed”, when actions of mowing, pruning, removal of dangerous or damaged trees, and partial removal of dead wood and dead branches are visible; as “managed” when past interventions are visible (e.g., thinning) and occasional pruning interventions are carried out; as “free evolution” when the urban forest is left without interventions to evolve naturally.

#### 4.1.1 Selected urban parks in Florence

“Parco delle Cascine” is the largest public park in Florence, covering approximately 160 hectares along the right bank of the Arno River [12,15]. Originally established as a Medici estate for agricultural purposes in the 16^th^ century, it was transformed into a public park in the 19^th^ century. Today, the park is managed by the Municipality of Florence and protected under national heritage laws. The park is composed of different vegetational zones, including open meadows, tree-lined avenues, and mixed woodlands. The dominant tree species include *Quercus ilex, Platanus hybrida, Pinus pinea*, and *Populus alba* [19]. The data presented here refers to a high forest – intensively managed – extended for about 16 ha and composed of mixed broadleaves, slightly dominated by *Tilia cordata* and *Celtis australis*. The surveyed urban forest covers about 0.5 % of the total urban park and 5 % of the area investigated.

“Giardino di Boboli” is a historic park in central Florence managed by the Uffizi Galleries and protected by UNESCO [7]. It extended for about 30 ha, representing the Italian-style garden with geometric layouts, sculptures, and fountains [13]. The park consists of different zones, including wooded areas dominated by *Quercus ilex*, structured gardens, meadows, and an exotic plant nursery. The main tree species include *Quercus ilex, Pinus pinea, Cupressus sempervirens*, and *Platanus orientalis*. The data presented here refers to a high forest – intensively managed – extended for about 23 ha and mainly dominated by *Q. ilex*, with the presence of monumental evergreen oaks, mainly dominated by *Q. ilex*. The surveyed urban forest covers about 2.67 % of the total urban park and 3.5 % of the area investigated.

“Villa Medicea della Petraia” was transformed into a Renaissance residence under the Medici family in the 16th century. The park is extended for about 30 ha in the hills of Florence, listed in the UNESCO sites of Medici villas, and governed by the Italian Ministry of Culture [5]. Three levels of a terraced Italian garden compose the park, the parterre, the nursery garden, and the “Prato della Figurina” garden. The main tree species include *Quercus ilex, Cupressus sempervirens, Pinus pinea*, and *Platanus orientalis*. Other species found in the park include *Cedrus deodara, Fraxinus ornus*, and *Acer campestre*, contributing to the site's rich botanical diversity. The wooded areas contain a mix of native and ornamental species, with a dense undergrowth of Mediterranean maquis with *Quercus ilex* and shrubs such as *Arbutus unedo, Laurus nobilis*, and *Myrtus communis*. The data presented here refer to a high forest – intensively managed – extended for about 19 ha and mainly dominated by *Q. ilex*. The surveyed urban forest covers about 2.67 % of the total urban park and 4.2 % of the investigated area.

“Villa Demidoff - Parco Mediceo di Pratolino” extended for about 120 ha, located in and managed by the Metropolitan City of Florence [20]. Originally designed in the 16^th^ century as a grand Medici estate, in the 19^th^ century, it was purchased by the Russian Demidoff family and transformed into a park, currently protected by regional heritage regulations. The park presents a diverse range of vegetational zones, including ancient woodlands, open meadows, and ornamental groves. The data presented here refer to a managed forest, extended for about 10 ha and mainly dominated by *Quercus ilex, Fagus sylvatica, Platanus orientalis*, and *Castanea sativa*. Notable ornamental species such as *Cedrus libani, Sequoiadendron giganteum*, and *Ginkgo biloba* add to the historical and botanical significance of the site. The surveyed urban forest covers about 0.67 % of the total urban park and 8 % of the investigated area.

#### 4.1.2 Selected urban parks in Rome

“Villa Borghese” is one of the public parks of the historic villas of the municipality of Rome, located in the city centre, and extended for about 80 hectares. The park is characterized by zones with different vegetational and management characteristics, showing both native and non-native species planted and established throughout the four centuries of its history. The dominant wooded areas consist primarily of evergreen oaks (*Quercus ilex*) but also include *Pinus pinea* and *Platanus hybrida* in structured plantations. Some sections feature *Celtis australis* and *Aesculus hippocastanum* along tree-lined avenues. The vegetation inside Villa Borghese covers about 72 % of the total park extension, 43 % of which are woods and mixed hardwoods, and 17 % tree-lined avenues [8]. The data presented here refer to a high forest – intensively managed – extended for about 2 ha and mainly dominated by *Q. ilex*, with the presence of monumental evergreen oaks. The surveyed urban forest covers about 1% of the total urban park and 40 % of area investigated. Villa Borghese – along with Villa Doria Pamphilj – is included in the Natura 2000 Network as a Special Area of Conservation (SAC IT6030052) for the conservation of three protected species: *Emys orbicularis* (code 1220), *Osmoderma eremita* (code 1084*) and *Cerambyx cerdo* (code 1088).

“Villa Ada Savoia” is the fourth largest public park in the municipality of Rome; it extends for 160 ha and originally served as a royal hunting reserve. The vegetation consists mainly of trees planted throughout history in different zones and some small semi-natural cores. The main tree species include *Quercus suber, Pinus pinea, Cedrus deodara*, and *Fraxinus ornus*. In particular, cork oak (*Q. suber*) forms dense woodlands, providing important habitats for local biodiversity. Isolated clusters of *Cedrus atlantica* and *Laurus nobilis* contribute to structural heterogeneity. The vegetation extension inside Villa Ada Savoia covers about 84 % of the total extension of the park of which 70 % are woods and 13 % lawns [8]. The data presented here refers to a high forest – intensively managed – extended for about 2.3 ha and mainly dominated by *Q. suber*, with the presence of monumental evergreen oaks. The surveyed urban forest covers about 0.5 % of the total urban park and about 36 % of the investigated area.

“Villa Doria Pamphilj” is the second-largest public park in the municipality of Rome and extends over 184 ha. originally the country estate of a noble family, it later became a hunting reserve. Several plant species have been planted over its four centuries of history, depending on the functions they fulfilled in the various areas of the park. The vegetation varies across different zones, with a dominance of *Quercus ilex, Pinus pinea*, and *Platanus hybrida*, alongside significant areas of mixed hardwoods. Vegetation extension inside Villa Doria Pamphilj covers about 90 % of the total surface area of the park, of which 19 % were woods and mixed hardwoods, 8 % tree-lined avenues, and 7 % “groups of trees” [8]. The data presented here refers to a high forest – intensively managed – extended for 2.6 ha and mainly dominated by *Q. ilex*, with the presence of monumental evergreen oaks interspersed with *P. pinea*. The surveyed urban forest covers about 0.43 % of the total urban park and 31 % of the investigated area.

“Riserva Naturale di Decima Malafede” is a protected area located mainly in the municipality of Rome, extending for 6145 hectares. Established in 1997, it includes agricultural lands and semi-natural woods, with mixed ownership, both public and private. Due to its extension, different phytoclimatic condition co-exists in this reserve, such as planitial vegetation, volcanic gorges with mesophilous vegetation, and rare shrubs with high biogeographic value [3]. The data here refers to an urban forest in free evolution, extended for about 1.2 ha and composed by mixed mesophil us and thermophilus broadleaves, mainly represented by *Quercus pubescens, Q. frainetto* and *Q. suber*. The surveyed urban forest covers about 0.01 % of the total urban park and 66.7 % of the investigated area.

#### 4.1.3 Selected urban parks in Campobasso

“Villa De Capoa” is a historic public garden in the centre of the municipality of Campobasso, extended for about 1.6 ha. Originally part of a 17^th^ century Franciscan botanical garden, it was later transformed into a neoclassical park and donated to the municipality in 1929, which still manages it as a public urban park. The park is an Italian-style garden with symmetrical pathways lined by evergreen hedges and enriched with statues, fountains, and historical monuments. The vegetation includes a diverse mix of native and ornamental tree species. The area is intensively managed, and dominant species are *Aesculus hippocastanum, Sequoiadendron giganteum, Cedrus libani, Cupressus sempervirens*, and *Picea abies*. Here, one of the monumental sequoias is registered in the National Register of Monumental Trees. The surveyed urban forest covers about 50 % of the total urban park.

“Rocca Monforte” is included within the Natura 2000 Network as a Special Area of Conservation (SAC IT7222125) for the conservation of habitats 6220* (sub-steppe pathways of grasses and annual plants of the Thero Brachypodietea), 6210* (semi-natural dry grassland formations and shrub-covered facies on limestone substrate (Festuco-Brometalia) with orchid blooms) and 8210 (limestone cliffs with chasmophytic vegetation). This site is included in the Monte Sant’Antonio complex, which is extended for about 12.87 ha and located on top of the medieval centre of the municipality of Campobasso. Four reforestation activities with *Pinus nigra* involved the area of Monte Sant’Antonio (i.e., 1961, 1976, 1979, 1981, Molise Region, Ministry of Agriculture and Forestry). Broadleaves expanded along years and the coppice area includes stands managed under lengthened rotations, currently visible. Data presented here refer to a part of this managed broadleaf forest extended for about 4.4 ha with *Quercus pubescens, Fraxinus ornus*, and *Acer campestre*, as dominant tree species and Mediterranean shrubs such as *Laurus nobilis* and *Arbutus unedo*. The surveyed urban forest covers about 6.22 % of the total urban park and 18.2 % of the area investigated.

“Parco dello Scarafone” is an urban green area of the municipality of Campobasso extended for about 25 ha, serving as an ecological corridor connecting the city centre, small urban parks of residential areas, and the surrounding natural landscapes. The park shows mixed ownership – both public and private – scarcely managed by local authorities (i.e., free evolution in most of the area) with no strict conservation restrictions. The area is characterized by a mix of open grasslands, reforested zones, and semi-natural woodlands. The data here refers to an area extended for about 1.4 ha with dominant tree species including *Quercus pubescens, Pinus nigra*, and *Fraxinus ornus*, with an understory composed of *Laurus nobilis* and *Crataegus monogyna*. The surveyed urban forest covers about 5.33 % of the total urban park and 57 % of the area investigated.

“Monte Vairano” is a natural forest included within the Natura 2000 Network as a Special Area of Conservation (SAC IT7222295) situated in the periurban area of the municipality of Campobasso, encompassing an area of diverse habitats with considerable ecological value. The site is extended for about 693 ha and governed by regional conservation measures aimed at preserving biodiversity and landscape. This area features dry grasslands (Festuco-Brometalia), and sub-Mediterranean oak forests (*Quercus frainetto*, and *Q. cerris*), the understory is mainly populated by *Crataegus monogyna, Hedera helix, Helleborus foetidus* [11]. The vegetation consists of mixed broadleaf forests and open meadows, supporting a high level of biodiversity. The data presented here refer to broadleaves forest extended for about 1.09 ha and mainly dominated by *Q. cerris*. This forest covers about 3.64 % of the total park and 73 % of the investigated area.

### 4.2 Experimental design and data collection

In each urban park of the investigated cities, data were collected within 15 circular plots with a radius of 13 m, distributed within a selected urban forest, featuring various forest types. All sites adhered to a systematically aligned sampling scheme and the sampling protocol follows the one proposed by Parisi et al. [14]. Information was gathered for all 180 sampling plots on living trees, tree-related microhabitats, and the different categories of deadwood. Living trees were sampled based on a minimum diameter at breast height ≥5 cm, and the following parameters were recorded, tree species, diameter at breast height, tree height, height-to-base of live crown, that is the height of the first living branch of the main crown, and tree-related microhabitats, according to the classification proposed by Kraus et al. [9]. The categories of deadwood include snags, standing dead trees, dead downed trees, coarse woody debris, and stumps. Snags are defined as standing dead trees without crowns, with a minimum height of 1.3 m, differing from standing dead trees, showing crowns with dead branches and twigs. Deadwood data were collected for a minimum diameter ≥5 cm, and a minimum length for coarse woody debris ≥1 m. Deadwood measurements were taken of their lengths, heights, and minimum and maximum diameters, when the second diameter of the snag was not manually collectable, it has been estimated by sight. The volume of living trees, standing and dead downed trees were calculated using the national double-entry volume equation [17], while the volumes of coarse woody debris, snags, and stumps were determined using the cone trunk formula [10].

## Limitations

Despite this data collection followed a systematically aligned sampling scheme, the 180 sampling plots were not placed along the entire extension of the selected urban parks. Instead, they were limited to freely accessible pre-selected urban forest zones responding to structural characteristics of interest to the authors (i.e., broadleaves urban forests, possibly enriched with monumental trees).

Furthermore, during the sampling period, some urban parks underwent maintenance interventions by public and private bodies responsible for their management. These interventions (e.g., pruning, removal of fallen trees and dead branches, and tree cuts) could have affected data collection and potentially altered an update and comparison of the data over time. However, in an urban environment, especially in recreational areas, people's health and safety need to be preserved as a priority, despite being areas of high ecological interest.

## Ethics Statement

The authors have read and agree with the ethical requirements for publication in Data in Brief and confirm that the current work does not involve human subjects, animal experiments, or any data collected from social media platforms.

## CRediT Author Statement

**Costanza Borghi:** Data curation, Investigation, Software, Original draft preparation, Writing- Reviewing and Editing; **Soraya Versace:** Data curation, Investigation, Software, Visualization**; Elena Di Pirro:** Visualization, Original draft preparation, Writing- Reviewing and Editing; **Davide Travaglini,** Methodology, Data curation, Validation**; Gherardo Chirici:** Validation, Supervision**; Bruno Lasserre:** Supervision, funding acquisition**; Marco Marchetti:** Supervision, Funding acquisition**; Giovanni D’Amico:** Data curation**; Elia Vangi:** Data curation, Software; **Saverio Francini:** Data curation, Software; **Marco Montella:** Data curation, Visualization, Investigation**; Giovanni Santopuoli:** Data curation; **Marco Ottaviano:** investigation; **Francesco Parisi:** Conceptualization, Data curation, Investigation, Methodology, Original draft preparation, Supervision, Writing- Reviewing and Editing.

## Data Availability

FigshareUrban and Periurban Forests of Italy: Structure, Tree-related Microhabitats, and Deadwood (Original data).
